# Holocene carbon dynamics at the forest–steppe ecotone of southern Siberia

**DOI:** 10.1111/gcb.13583

**Published:** 2016-12-28

**Authors:** Anson William Mackay, Alistair W. R. Seddon, Melanie J. Leng, Georg Heumann, David W. Morley, Natalia Piotrowska, Patrick Rioual, Sarah Roberts, George E. A. Swann

**Affiliations:** ^1^ Environmental Change Research Centre Department of Geography UCL London WC1E 6BT UK; ^2^ Department of Biology and Bjerknes Centre for Climate Research University of Bergen PO Box 7803 Bergen N‐5020 Norway; ^3^ NERC Isotope Geosciences Facilities British Geological Survey Nottingham NG12 5GG UK; ^4^ Centre for Environmental Geochemistry University of Nottingham Nottingham NG7 2RD UK; ^5^ Steinmann Institute of Geology, Mineralogy and Paleontology University of Bonn Nussallee 8 53115 Bonn Germany; ^6^ Department of Radioisotopes Institute of Physics – CSE Silesian University of Technology Konarskiego 22B 44‐100 Gliwice Poland; ^7^ Key Laboratory of Cenozoic Geology and Environment Institute of Geology and Geophysics Chinese Academy of Sciences PO Box 9825 Beijing 100029 China; ^8^ School of Geography University of Nottingham University Park Nottingham NG7 2RD UK

**Keywords:** abrupt climate change, carbon, forest–steppe ecotone, Holocene, Lake Baikal, palaeolimnology, permafrost

## Abstract

The forest–steppe ecotone in southern Siberia is highly sensitive to climate change; global warming is expected to push the ecotone northwards, at the same time resulting in degradation of the underlying permafrost. To gain a deeper understanding of long‐term forest–steppe carbon dynamics, we use a highly resolved, multiproxy, palaeolimnological approach, based on sediment records from Lake Baikal. We reconstruct proxies that are relevant to understanding carbon dynamics including carbon mass accumulation rates (CMAR; g C m^−2^ yr^−1^) and isotope composition of organic matter (*δ* ^13^ C_TOC_). Forest–steppe dynamics were reconstructed using pollen, and diatom records provided measures of primary production from near‐ and off‐shore communities. We used a generalized additive model (GAM) to identify significant change points in temporal series, and by applying generalized linear least‐squares regression modelling to components of the multiproxy data, we address (1) What factors influence carbon dynamics during early Holocene warming and late Holocene cooling? (2) How did carbon dynamics respond to abrupt sub‐Milankovitch scale events? and (3) What is the Holocene carbon storage budget for Lake Baikal. CMAR values range between 2.8 and 12.5 g C m^−2^ yr^−1^. Peak burial rates (and greatest variability) occurred during the early Holocene, associated with melting permafrost and retreating glaciers, while lowest burial rates occurred during the neoglacial. Significant shifts in carbon dynamics at 10.3, 4.1 and 2.8 kyr bp provide compelling evidence for the sensitivity of the region to sub‐Milankovitch drivers of climate change. We estimate that 1.03 Pg C was buried in Lake Baikal sediments during the Holocene, almost one‐quarter of which was buried during the early Holocene alone. Combined, our results highlight the importance of understanding the close linkages between carbon cycling and hydrological processes, not just temperatures, in southern Siberian environments.

## Graphical abstract

The forest–steppe ecotone in southern Siberia is highly sensitive to climate change; global warming is expected to push the ecotone northwards, at the same time resulting in degradation of the underlying permafrost. To gain a deeper understanding of long‐term forest–steppe carbon dynamics, we use a highly resolved, multiproxy, palaeolimnological approach, based on sediment records from Lake Baikal. Generalized additive models identify significant change points in temporal series, which we relate to abrupt sub‐Milankovitch scale events. About 1.03 Pg of C has been buried in Lake Baikal sediments since the start of the Holocene, with almost one‐quarter of budget being buried within the first few thousand years.

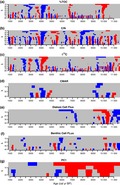

## Introduction

Permafrost is highly vulnerable to global warming and in recent decades has experienced temperature increases of up to 3 °C, with multiple, complex impacts on vegetation, hydrology and the biogeochemical cycling of carbon (Vaughan *et al*., 2013). Sporadic‐isolated permafrost regions are especially at risk, including those in southern Siberia–northern Mongolia, from degradation through warming, human impact and increased wildfires (Sharkuu, 1998; Romanovsky *et al*., 2010; Zhao *et al*., 2010; Törnqvist *et al*., 2014). Globally, permafrost contains one of the largest pools of organic carbon, and warming ultimately results in the release of this carbon pool to the atmosphere via microbial degradation (Schuur *et al*., 2008). Old organic carbon liberated from melting permafrost may also be exported to headwater streams and rivers as dissolved organic carbon (DOC; Spencer *et al*., 2015). In central Siberia, large amounts of DOC are transported from catchments into lakes, especially via rivers at more southerly latitudes where sporadic and isolated permafrost is extensive (Prokushkin *et al*., 2011).

Over long timescales, the nature of carbon release from permafrost soils is rather uncertain (Schuur *et al*., 2008), but one potential, underutilized tool for understanding how climate change has influenced carbon dynamics is by lacustrine sediment records of organic geochemistry. These records reflect long‐term interactions between lakes and their catchments (Anderson, 2014), especially regions underlain by permafrost (Vonk *et al*., 2012). Lakes in general act as an important control on the global carbon cycle, despite occupying only a small percentage of the surface of the earth. Carbon burial to the bottom of lakes is substantial, especially considering the quantities of sediment that have accumulated since the end of the last glaciation, which likely represents more than two‐fifths (42 Tg C yr^−1^) of the amount of organic carbon buried in ocean sediments (c. 100 Tg C yr^−1^; Dean & Gorham, 1998).

Within lake sediments, a number of different indicators can be used to record the responses of carbon cycling to extrinsic drivers such as climate. For example, sedimentary total organic carbon (TOC) provides a first‐order estimate of the amount of bulk organic matter that escapes remineralization during sedimentation (Meyers & Lallier‐Verges, 1999). However, TOC is sensitive to changes in sediment accumulation rates, and so arguably a better estimate of organic carbon burial is achieved through the calculation of carbon burial (or mass accumulation) rates (CMAR; g C m^−2^ yr^−1^; Meyers & Teranes, 2001) which are closely associated with the delivery of allochthonous carbon to lakes (e.g. Watanabe *et al*., 2009; Hyodo & Longstaffe, 2011; Moy *et al*., 2011). Sources of organic carbon sequestered into lake sediments may be further discriminated through their carbon isotope composition (*δ* ^13^C_TOC_) and TOC/total nitrogen (C/N) ratios (Leng & Marshall, 2004). Lake sediment records can also reveal major vegetation changes in the forest–steppe ecotone (through pollen analysis, e.g. Bezrukova *et al*., 2010; Iglesias *et al*., 2014), as well as shifts between primary producers (e.g. diatoms), linked to climate variability (Weckström *et al*., 2014). Multiproxy palaeolimnology is a powerful approach to gain deep insight into ecosystem dynamics in permafrost regions over long timescales.

One of the most important ecosystems in southern Siberia is Lake Baikal and its catchment. It is the world's largest lake by volume, but it is also the deepest and oldest lake, with sedimentary records spanning at least 20 million years. Its catchment spans almost 450 000 km^2^, from the southern limit of the boreal forest into the steppe regions of northern Mongolia. About 80% of Baikal's catchment belongs to its largest tributary, the Selenga River, which alone accounts for over half of all river input into the lake. Catchment permafrost is extensive – continuous and discontinuous permafrost dominates the east and west portions of the basin (ca. 30%), while sporadic and isolated permafrost dominates the south (Sharkuu, 1998; Törnqvist *et al*., 2014). Annual air temperature trend maps for the past 50 years show southern Siberia to be experiencing some of the largest increases globally (Jones *et al*., 2012), threatening vulnerable carbon pools including permafrost (Schuur *et al*., 2008; Romanovsky *et al*., 2010) and the hemiboreal forests (DeLuca & Boisvenue, 2012; Wu *et al*., 2012). Lake Baikal itself is also responding to regional warming; surface water temperatures and summer stratification have increased in recent decades (Hampton *et al*., 2014), while ice cover duration and thickness have declined (Todd & Mackay, 2003). Its long sedimentary record contains an estimated 4500 Pg of organic carbon, more than 400 times that contained in its catchment soils (Alin & Johnson, 2007), which is essentially locked away permanently. More relevant for understanding contemporary lake‐catchment interactions is the amount of organic carbon sequestered since the last deglaciation, which is currently unknown, and the role that climate may have played in this process. Understanding how climate change influenced carbon dynamics in the past has the potential to provide important insights for understanding how global warming may influence lake‐catchment carbon dynamics into the future.

Here, we apply a palaeolimnological, multiproxy approach to understand Holocene carbon dynamics in the Baikal–Selenga catchment at a multidecadal resolution. Global temperatures during the early Holocene were at least as warm as today (Marcott *et al*., 2013), and rates of permafrost warming during the early Holocene were also comparable to rates estimated for present day (Anisimov *et al*., 2002). Therefore, comparisons between early and late Holocene periods may provide useful insights into understanding long‐term carbon dynamics at the forest–steppe ecotone. The Holocene also experienced several centennial‐scale abrupt events (Mayewski *et al*., 2004; Wanner *et al*., 2014), such as the 8.2 kyr cold event (Kleiven *et al*., 2008) and the 4.1 kyr arid event (Cullen *et al*., 2000) but the extent to which these can influence Holocene carbon dynamics in permafrost regions remains unknown. The multidecadal, multiproxy data set offered in this study has potential to provide several key insights into carbon dynamics in a climate‐sensitive, permafrost region. To analyse these data, we use a generalized additive modelling version of a SiZer analysis (Chaudhuri & Marron, 1999; Korhola *et al*., 2000) for pinpointing significant points of change in the different temporal series and use generalized least‐squares regression to investigate how key components of carbon cycling in the lake respond to long‐term changes in climate variability. The data set and methods we have developed and applied in this study present a unique opportunity to address three principal questions:

1. What are the factors influencing carbon dynamics during early Holocene warming, and how do they compare to the late Holocene?
2. How did carbon dynamics respond to abrupt sub‐Milankovitch scale (e.g. 8.2 and 4.1 kyr) events?
3. What is the carbon storage budget for Lake Baikal during the Holocene, and how does this compare with other lakes?

## Materials and methods

### Study site

The Lake Baikal basin is situated in one of the world's most continental regions; summers are short, warm and wet, while winters are long, dry and cold. Summer rainfall stems from the progression of cyclones moving in from west Siberia. In autumn, cold Arctic air intrudes from the Kara Sea to central Asia, which leads to the growth of the Siberian High, a high‐pressure cell which intensifies during winter, and leads to cold air passing into Asia (Gong & Ho, 2002) influencing the intensity of the East Asian Winter Monsoon (EAWM; Wu & Wang, 2002).

**Figure 1:**
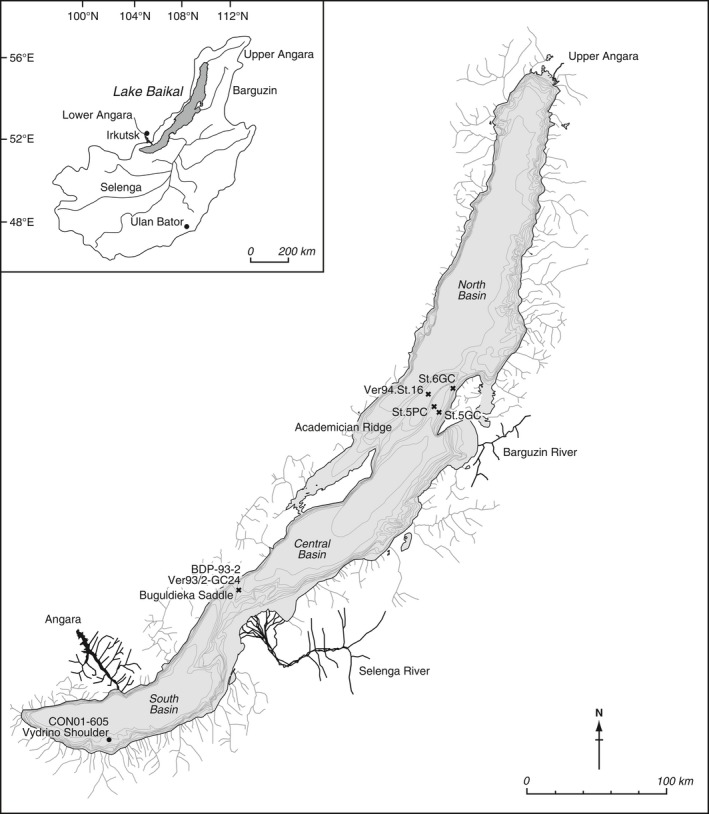
Map of Lake Baikal and its catchment, with locations of the different cores mentioned or utilized in this study highlighted.

The Vydrino Shoulder (51.58° N, 104.85° E) is an isolated high in the south basin of Lake Baikal (Fig. 1). It forms an upper‐ to mid‐slope, underwater terrace of mostly fine‐grained sediments, free from turbidites and unaffected by bottom‐water currents which can cause sediment focussing (Charlet *et al*., 2005). The Shoulder sits off‐shore from several major south basins tributaries (including the Snezhnaya and Vydrinaya rivers, which have their source in the neighbouring Khamar‐Daban mountain range) and is approximately 130 km from where the Selenga River enters Lake Baikal. Sidescan sonar mosaics and seismic data (Charlet *et al*., 2005) show the upper terrace sediments to be relatively undisturbed by tectonic activity and reworking and are therefore suitable for Holocene reconstructions. In the summer of 2001, a suite of cores was extracted from an off‐shore ridge crest location of continuous sedimentation (>600 m water depth) including a box core (CON01‐605‐5) and a piston core (CON01‐605‐3). During retrieval, the upper 12.5 cm of surface sediment was lost from the box core, representing the past c. 800 years. To provide context for carbon dynamics related to recent regional warming, carbon mass accumulation rates were calculated for the past 50 years from a UWITEC gravity core (BAIK13‐7) taken in 2013 to the west of CON01‐605 cores. Full details of the various core codes, their locations and relevant analyses are given in Table 1.

**Table 1:** Location of sediment cores investigated in this study and their analyses undertaken

Core code	Type	Lat.	Long.	Water depth	Core length	Analyses
CON01‐605‐3	Piston	51.5849	104.8548	675 m	10.45 m	DBD; diatoms
CON01‐605‐5	Box	51.5835	104.8518	665 m	2.50 m	^14^C; *δ* ^13^C_TOC_; TOC; C/N; CMAR; pollen
BAIK13‐7	Gravity	51.5683	104.5286	1080 m	0.47 m	DBD; TOC; CMAR

### Dating

**Figure 2:**
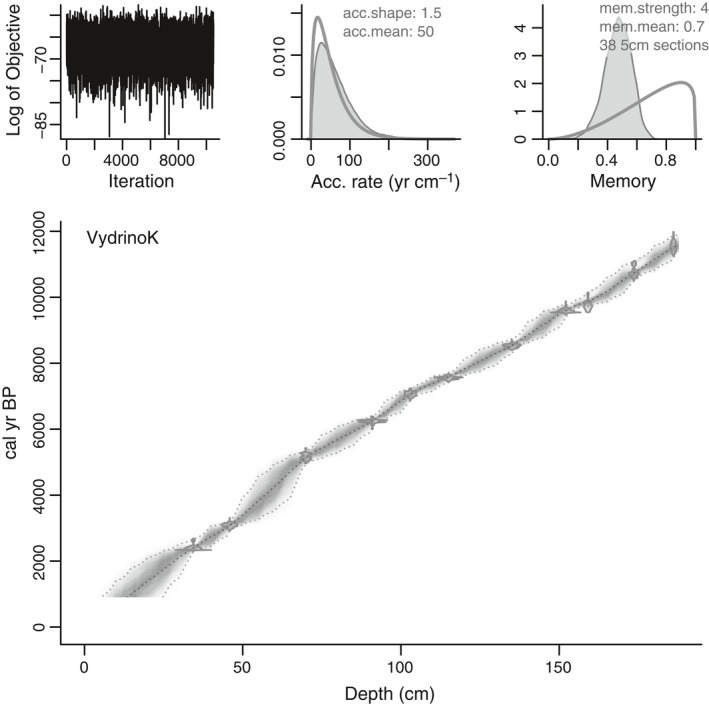
‘Bacon’ Age‐depth model (Blaauw & Christen, 2011) for Vydrino box core (CON01‐605‐05) of radiocarbon AMS dates calibrated using IntCal13 radiocarbon calibration curve (Reimer *et al*., 2013).

Radiocarbon dates were obtained by accelerated mass spectrometry (AMS) from pollen and spore concentrates from twelve box core (CON01‐605‐5) samples (Piotrowska *et al*., 2004; Table S1). All radiocarbon dates were calibrated using IntCal13 radiocarbon calibration curve (Reimer *et al*., 2013). Age‐depth modelling was done using ‘Bacon2.2’, allowing for variable sediment accumulation rates (Blaauw & Christen, 2011; see Fig. 2). The core was divided into 38 five‐cm sections, and prior parameters used for calculations were 50 years per cm for accumulation rate with gamma distribution shape 1.5, and default settings for memory (see Fig. 2). The results of Markov chain Monte Carlo iterations plotted in the upper left corner of Fig. 2 indicate good performance of the model. Sediment samples from BAIK13‐7 were dated using ^210^Pb analyses by nondestructive gamma spectrometry. Chronologies were calculated using the CRS (constant rate of ^210^Pb supply) dating model, after corrections were made for the effect of self‐absorption of low energy gamma rays within samples (Appleby, 2001).

### Palaeoecology

Pollen and diatom analyses were undertaken on two different cores extracted from the Vydrino Shoulder (Table 1). Pollen data were analysed at 10‐mm intervals from the box core CON01‐605‐5 and were used to represent long‐term vegetation changes in the surrounding landscape. Pollen was counted at magnifications of 400–600x, with critical identifications made at 1000x (see Demske *et al*., 2005 for full details). Here, we report on total arboreal pollen (AP) and *Pinus sylvestris* pollen (PynSylv; Scots Pine) as indicators of forest dynamics. A steppe–boreal forest index was also calculated as [(*Artemisia*+ chenopods +*Ephedra*)/AP]*100 (Traverse, 1998 in Bezrukova *et al*., 2005).

We used a principal components analysis (PCA) on the pollen data to summarize long‐term vegetation trends in around the lake (Fig. S1). The pollen percentage data were Hellinger‐transformed prior to analysis. For all subsequent analyses, we multiplied PC1 by ‐1 so that increases in the values of PC1 reflect expansion of boreal forest.

Diatoms were analysed at 5 mm resolution from the piston core (CON01‐605‐3) and represent a proxy for the main contributions of primary productivity within the lake. For each sample, at least 300 valves were counted using oil immersion phase‐contrast light microscopy at ×1000 magnification. Diatom cell fluxes (total and benthic; cm^−2^ yr^−1^ ×10^6^) were estimated by the addition of divinylbenzene microspheres (Battarbee & Kneen, 1982), together with calculated sedimentation rates (cm yr^−1^).

### Isotope geochemistry

Isotope geochemistry was undertaken on the box core (CON01‐605‐5) on contiguous 5‐mm samples and was used to understand different components of carbon cycling (Leng & Marshall, 2004). Sediments were placed in 5% HCl to remove any CaCO_3_ (assumed negligible) then washed over Whatman 41 filter papers with deionized water and dried at 40 °C in a drying cabinet. When dry, samples were ground to a fine powder and stored in glass vials. Carbon isotope ratios (*δ* ^13^C_TOC_), percentage total organic carbon (%TOC) and percentage total nitrogen (%TN; used to calculate C/N) were analysed during combustion in a Carlo Erba 1500 online to a VG Triple Trap and dual‐inlet mass spectrometer. *δ* ^13^C_TOC_ values were converted to the V‐PDB scale using a within‐run laboratory standard calibrated against NBS‐19 and NBS‐22, with C/N ratios calibrated against an Acetanilide standard. Replicate analysis of sample material indicated a precision of ±0.1‰ for *δ* ^13^C_TOC_ and ±0.1 for C/N. %TOC was also calculated for the past 50 years on BAIK13‐7 sediments, using the methods outlined above.

### Carbon mass accumulation rates

Only sediment samples from the piston core (CON01‐605‐3) were routinely analysed for wet densities and % dry weight at 105 °C, from which dry bulk density (DBD) values could be calculated (Table 1). Therefore, mean piston‐core DBD values for 100‐year intervals during the Holocene were calculated for the piston core. These were used alongside mean %TOC values for 100‐year intervals of the Holocene box core (CON01‐605‐5) to derive organic matter densities (g cm^−3^). Using the Box core calibrated age model (cm yr^−1^), organic carbon mass accumulation rates (CMAR; g C m^−2^ yr^−1^) were calculated on the centennial‐scale averages of %TOC and DBDs. CMAR were also calculated for the past 50 years using %TOC, DBD and sediment accumulation rates calculated for BAIK13‐7.

### Statistical modelling of the Vydrino data sets

Ecological dynamics are subject to modes of variability across a variety of temporal scales (Jackson & Overpeck, 2000) and so one curve may not be sufficient to capture the complete components of variability within a temporal series. Therefore, for a full appreciation of the long‐term dynamics of carbon cycling in Lake Baikal over the Holocene approaches that can take multiple temporal dynamics into account are needed. SiZer analyses (e.g. Chaudhuri & Marron, 1999) can capture such dynamics, by identifying significant trends at different modes of variability. In this study, we developed our own version of a SiZer analysis and applied it to each of the variables using generalized additive modelling (GAM; Wood, 2006). Our method allows temporal autocorrelation to be fitted within each model, which should result in more conservative tests when testing for significant trends (e.g. Park *et al*., 2004).

To develop our GAM SiZer method, we used the following procedure combining functions within the package mgcv (Wood, 2006) and a script developed by Simpson (2014) in r (R Development Core Team, 2016) on each of the variables:

1. fix the smoothing parameter *k* to a given value using the option in the smoothing term ‘fx = TRUE’;
2. test for temporal autocorrelation in the residuals in the model assuming an exponential decay function (e.g. Seddon *et al*., 2014);
3. refit the GAM with an appropriate variance–covariance matrix reflected by the temporal autocorrelation using the stable multiple smoothing parameter estimation method (Wood, 2004);
4. test for the significance of the slope of the GAM spline using a simultaneous confidence interval method described by Simpson (2014);
5. identify which periods contain significantly increasing/decreasing trends;
6. repeat for different values of *k* (*k *=* *5, 10, …, *k*_max_);
7. map the time periods of significantly increasing or decreasing trends in a SiZer plot, with positive trends identified in red and negative trends identified in blue.

The value *k*_max_ is dependent on sample size, and the different sample resolution and temporal structures of our data sets mean that overfitting may be an issue at higher values of *k*. Therefore, to estimate the maximum value of *k,* we used the ‘gam.check()’ function in the mcgv package to test whether the smoothing basis dimension for a GAM spline was too high. This command employs a test to compare the residual variance of a model fit with the difference of residuals between neighbours and then randomly reshuffles the residuals 1000 times to find a null distribution of variance differences (see help file for gam.check() function in mgcv, Wood, 2006). For each data set, our value *k*_max_ was selected according to when the variance differences moved above *P *=* *0.05 from the null distribution. Information on the data transformations used (to enable our models to be run using Gaussian error distributions, the *k*_max_ values and the mean and median sample resolutions for the different data sets) is provided in Table S2.

The GAM SiZer methodology presented here is useful for identifying periods of major change within individual temporal series, but our multiproxy study design also means that we were able to use statistical modelling to investigate whether longer‐term changes in organic geochemistry were linked to changes in climate. A piecewise linear regression revealed a break point in PC1 axis representing long‐term forest–climate responses at c. 6051 ± 241 cal years bp (Fig. S2). Therefore, we split the data into early Holocene (EH, 11.6–6.1 kyr) and late Holocene (LH, 6.1–0.8 kyr) periods and ran linear regressions to check for relationships between long‐term landscape/climate changes and organic geochemistry. Since the CMAR data set had a different age model to the pollen data, the pollen data were linearly interpolated to the sample ages of the CMAR data set. We then used a generalized least‐squares regression to test for relationships between climate and the different within‐lake proxies for the two time periods. We checked for the presence of temporal autocorrelation in the residuals and then fitted a new model assuming exponential decay function to describe the degree of association between samples if required (e.g. Seddon *et al*., 2014). The models including autocorrelation were compared using the Akaike information criterion (AIC), and the best model (lowest AIC) was used to interpret drivers of the changes of carbon cycling over time.

## Results

Sediment sample ages calculated on modelled weighted means show that the box core sediments were deposited between c. 11.6 and 0.8 cal kyr bp (Fig. 2). Sediment accumulation rates (SAR) range between 30.9 and 9.8 cm kyr^−1^ (mean 16.3 cm kyr^−1^), with peak values calculated at 9.8 kyr bp. Thereafter, SAR decline to a low between 4.5 and 4.4 kyr bp.

**Figure 3:**
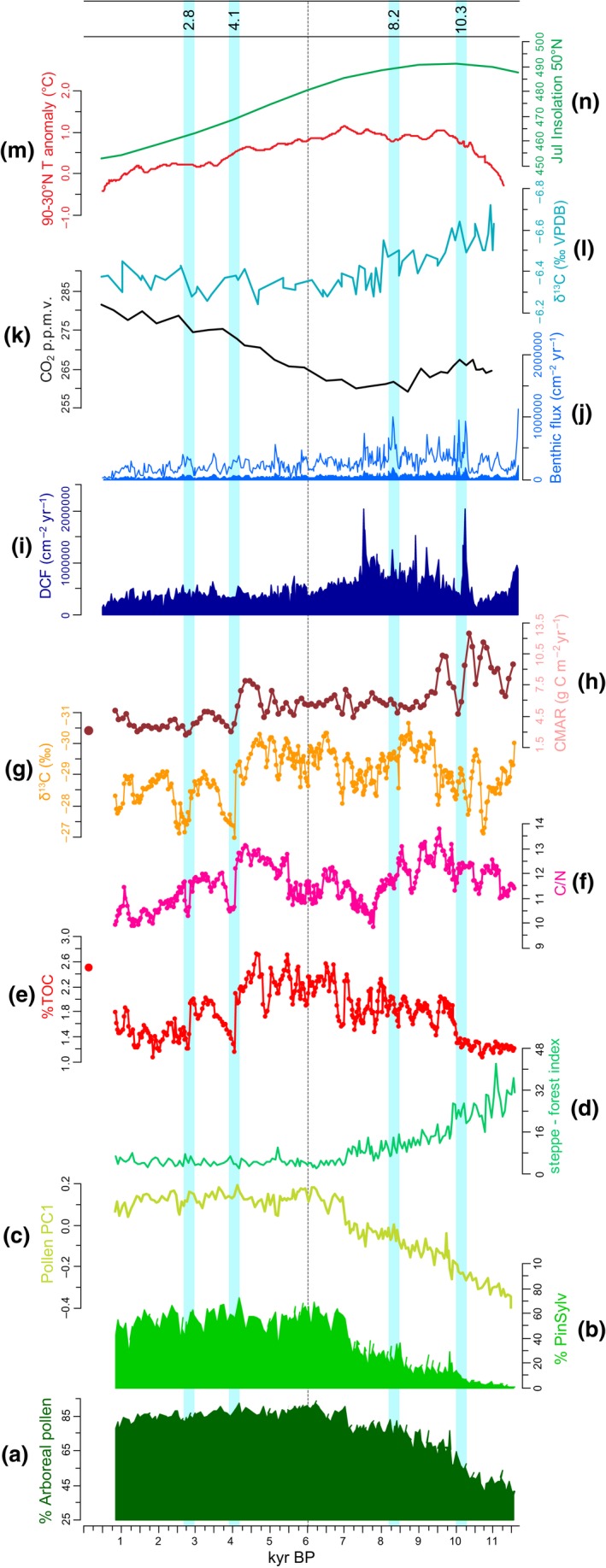
Multiproxy data determined for Holocene sediments from the Vydrino Shoulder, Lake Baikal. Vegetation (a–d) and organic geochemistry data (e–h) are from Vydrino Shoulder core CON01‐605‐5. Diatom data (i–j) are from Vydrino Shoulder core CON01‐605‐3. (a): % Arboreal pollen (b): *Pinus sylvestris* pollen (%PinSylv); (c): Pollen PC1 scores; (d): steppe–forest index; (e): total organic carbon (%TOC); (f): total organic carbon/total organic nitrogen ratios (C/N); (g): *δ* ^13^ C_TOC_ (‰); (h): carbon mass accumulation rates (CMAR; g C m^−2^ yr^−1^) in 100‐year bins; (i): diatom cell fluxes (DCF cm^−2^ yr^−1^ x10^6^) from CON01‐605‐3; (j): benthic diatom fluxes (filled silhouette) with ×5 exaggeration to see fluxes in detail (empty silhouette); (k): CO_2_ data (p.p.m.v.) from Dome C ice core (Flückiger *et al*., 2002); (l): *δ* ^13^C ice core records Dome C ice core (Elsig *et al*., 2009); (m): mean Northern Hemisphere temperature stack records for 60° latitude bands (30° N – 90° N; Marcott *et al*., 2013); (n): July insolation 50° N (W m^−2^; Berger & Loutre, 1991). The horizontal dotted line at 6.1 kyr bp marks significant change in PC1 identified by break point analysis. Light blue zones denote abrupt reversal events at c. 10.3, 8.2, 4.1 and 2.8 kyr bp.

**Figure 4:**
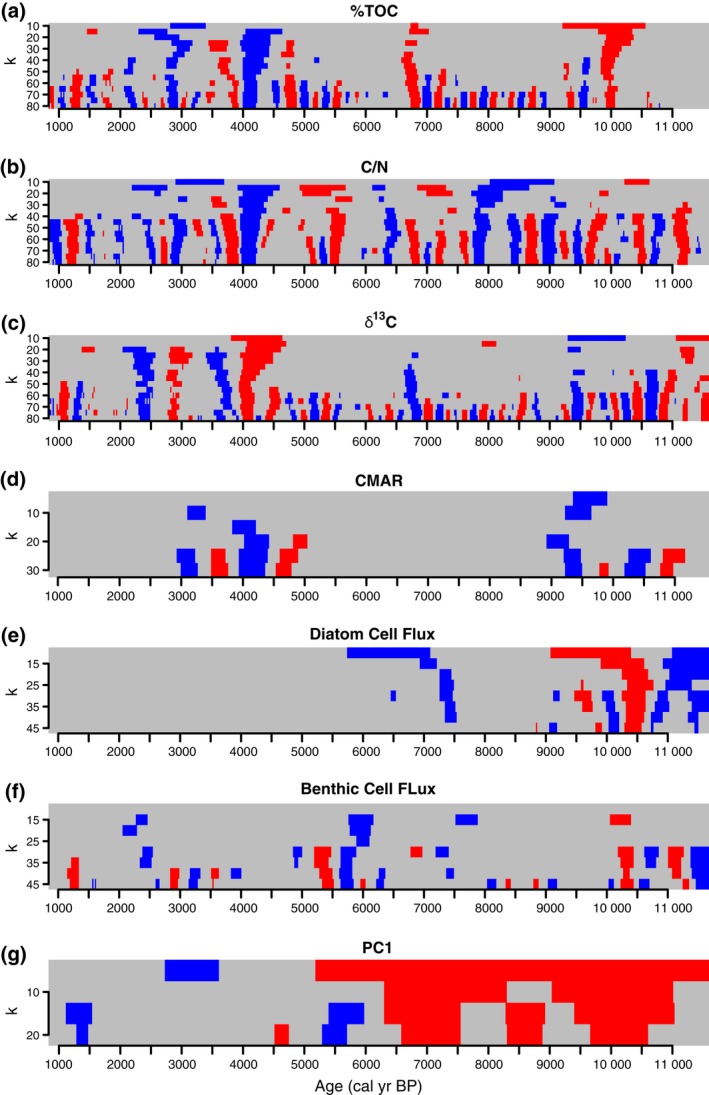
Individual SiZer plots from our GAM SiZer analyses. Grey areas are periods of nonsignificant change, while blue and red periods show periods of significant decreasing/increasing change, respectively.

The stratigraphic data are presented in Fig. 3 and the individual SiZer plots in Fig. 4. Assessment of the SiZer plots help to identify key events and trends in the different proxy profiles. Steppe communities were prevalent in the watershed of Lake Baikal during the early Holocene but declined abruptly at c. 10 kyr bp, before gradually declining to very low values at c. 6.1 kyr bp (Fig. 3d). Pollen from steppe vegetation remained a small but persistent feature of the record for the remainder of the Holocene. *Pinus sylvestris* (Scots pine) was virtually absent, but became dominant (i.e. over 50% total land pollen; TLP) by 7.0 kyr bp (Fig. 3b). For the remainder of the record, tree pollen was above 80% TLP. The first principal component (PC1) of the pollen data explained 73.3% of the total variance of the data set (significant by comparison to the broken stick model, Line & Birks, 1996) and was dominated by a gradient between cold‐adapted species such as dwarf birch and the eurythermic Scots Pine (Fig. S1). In general, there was a significant long‐term increasing trend in PC1 from the start of the Holocene to become more stable during the late Holocene at lower values of *k* (Figs 3c and 4g).

Total diatom cell fluxes (DCF) ranged from c. 0.04 to 2.03 million cells cm^−2^ yr^−1^ (Fig. 3i). Fluxes were especially significant before 10 kyr bp (Fig. 4e). A final significant decline in DCF was observed at 7.5 kyr bp (Fig. 4e), with no further significant variability for the remainder of the Holocene. In contrast, the fluxes of benthic diatom cells showed more significant variability, particularly at higher frequencies (i.e. higher values of *k*) for much of the Holocene (Fig. 4f). For example, while there were large oscillations in benthic diatom fluxes before c. 10 kyr bp, we also observed significant flux declines at c. 7.5 and 5.5 kyr bp (Figs 3j and 4f). Mean benthic flux rates for the complete Holocene was 56 000 cells cm^−2^ yr^−1^, or c. 10% of mean diatom cell fluxes, highlighting the overall dominance of the planktonic contribution to diatom productivity in this core.

Total organic carbon values were very low during the initial stages of the early Holocene (11.6–10.1 kyr bp; mean 1.2%), followed by a significant increase in %TOC values at 10.0 kyr (Figs 3e and 4a), reflecting a step‐like shift into increasingly higher Holocene values. In general, three other major periods of change were identified by SiZer analysis: an increase in %TOC at 6.8 kyr bp and declines in %TOC at 4.1 kyr bp and 2.8 kyr bp (Fig. 4a), reflecting local minima (Fig. 3e). In BAIK13‐7, TOC in the uppermost sediments deposited during the past 50 years reached 2.5% (Roberts, 2016), the highest values since 4.7 kyr bp, and some of highest values for the whole Holocene. Sedimentary *δ* ^13^C_TOC_ and C/N ratios were also highly variable and show similar patterns to %TOC. For example, sedimentary *δ* ^13^C_TOC_ ranges between −30.7 and −27.0‰ (mean −29.03 ‰), with high frequency oscillations found throughout the record (Fig. 3g), and significant periods of change around 9.4, 7.4, 4.1, 3.6, 2.8 and 2.4 kyr bp (Fig. 4c). C/N ratios fluctuate between 9.9 and 13.8 (mean = 11.6; Fig. 3f). Abrupt and significant declines are observed at 7.8, 4.1 and 2.8 kyr bp (Figs 3f and 4b).

Organic carbon mass accumulation rates were highest during the early Holocene (11.6–9.0 kyr bp; Fig. 3h). The SiZer analysis revealed this was also a major period of variability, particularly at higher frequencies (Fig. 4d). For example, peak values of 12.5 g C m^−2^ yr^−1^ were observed at 10.4 kyr bp before they declined rapidly to c. 4.8 g C m^−2^ yr^−1^ at 10.1 kyr bp. A further significant decline was observed between 9.5 and 9.3 kyr bp. Between c. 4.5 and 4.0 kyr CMAR exhibited a significant decline from 7.9 g C m^−2^ yr^−1^ to 3.1 g C m^−2^ yr^−1^. For much of the late Holocene, CMAR remained low <5 g m^−2^ yr^−1^ with a distinct minimum at 2.8 kyr bp. Mean Holocene CMAR was 5.9 g C m^−2^ yr^−1^. During the past five decades, mean CMAR in BAIK13‐7 were only c. 3 g C m^−2^ yr^−1^ (Fig. 3h).

**Figure 5:**
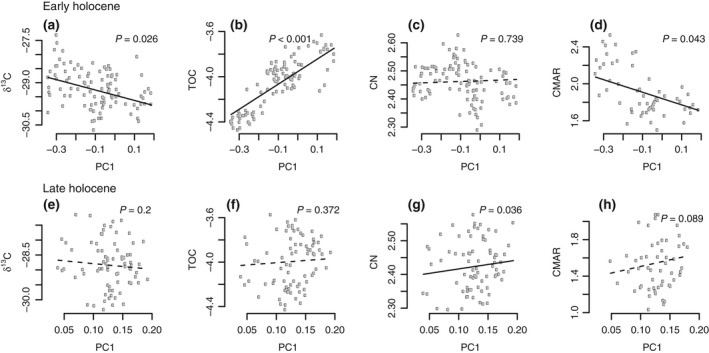
Modelled relationships between PC1 scores and organic geochemistry for early (a–d) and late (e–h) periods. Solid line indicates a significant relationship, *P* = 0.05.

Modelled PC1 (i.e. the cold‐adapted/eurythermic gradient in the pollen data) relationships with organic geochemistry highlight stronger responses during the early Holocene (Fig. 5a–d) than late Holocene (Fig. 5e–h). Although the most significant (positive) relationship was between %TOC and PC1 during the early Holocene (Fig. 5b), when expressed as burial rates, the strength of the relationship between PC1 and C declined and was negative (Fig. 5d). A significant negative relationship between PC1 and *δ* ^13^C_TOC_ was also observed (Fig. 5a), although these relationships were not significant following a sequential Bonferroni correction. In contrast, the only significant relationship found during the late Holocene was between PC1 and C/N values which was also removed once a sequential Bonferroni correction was applied (Fig. 5g). Given that the sequential Bonferroni corrections can be overly conservative and make it difficult to observe multiple significant relationships in noisy (e.g. ecological) data (Moran, 2003), we attempt to ascribe a physical basis to patterns of variability related to uncorrected significant models in the discussion where possible.

## Discussion

Overall concentrations of sedimentary organic carbon in Lake Baikal are low due to high remineralization rates in the water column (Müller *et al*., 2005) and poor burial efficiency (Maerki *et al*., 2006; Sobek *et al*., 2009, 2014). Burial efficiency is as poor in Lake Baikal as it is in the oceans because of low sediment accumulation rates leading to very high oxygen exposure times (between 10 and over 1000 years, Sobek *et al*., 2009). Moreover, organic carbon is dominated by autochthonous production (phytoplankton contribute approximately 90% of organic matter in Lake Baikal, with less than 10% delivered from the catchment (Votintsev *et al*., 1975)) which makes it less resistant to oxidation (Sobek *et al*., 2009). Recently buried organic carbon is also subject to substantial postdepositional degradation, and while this may impact the very recent measurements from BAIK13‐7 (discussed below), the impact on our older sediments of >800 years will likely be very minor (Sobek *et al*., 2014). Previous multiple‐lake studies are usually based on single cores taken from central, deep locations, regions that are also subject to sediment focussing, which can result in carbon burial rates higher than expected. While some studies have made corrections for sediment focussing (e.g. Anderson *et al*., 2014; Heathcote *et al*., 2015), others have not (e.g. Dong *et al*., 2012). Crest environments on isolated and interbasin highs (i.e. the Vydrino Shoulder and the Academician Ridge) are not subject to sediment focussing, so no corrections were needed in this study.

### What are the factors influencing carbon dynamics during early Holocene warming and how do they compare to the late Holocene?

#### Early Holocene

**Figure 6:**
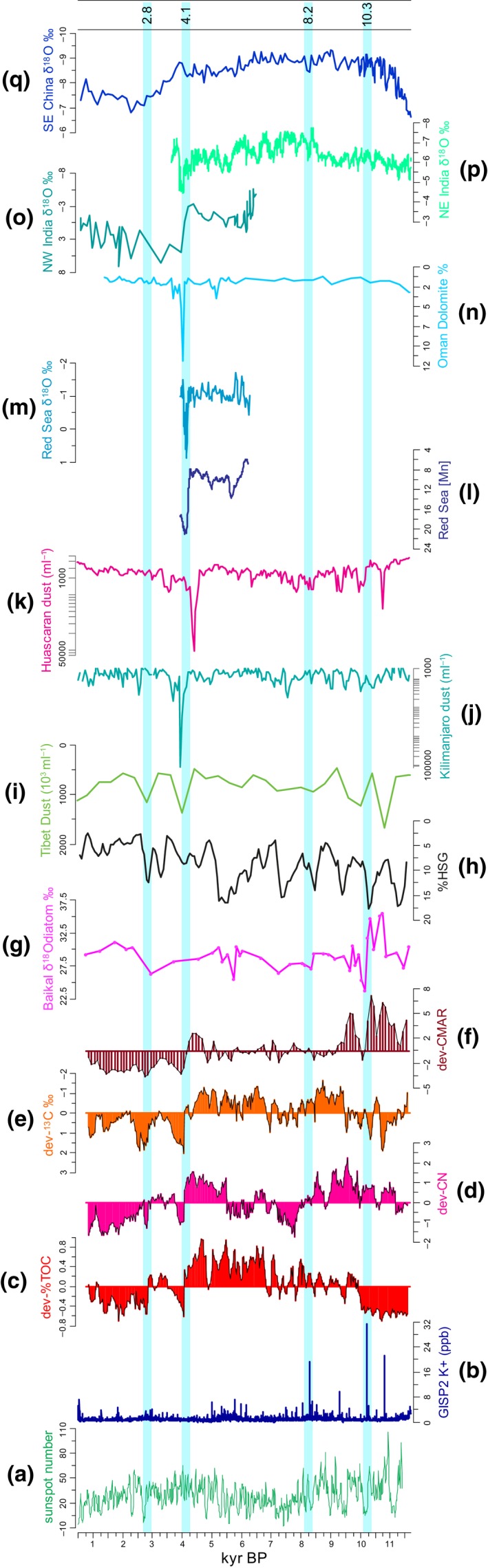
Multiarchive data plotted alongside ‘deviations from mean’ values of organic geochemical records (c–f) from Vydrino Shoulder core CON01‐605‐5. (a): Sunspot numbers (Solanki *et al*., 2004); (b): K^+^ ion concentrations (ppb) from GISP2 D core (Mayewski *et al*., 1997); (c): total organic carbon (%TOC); (d): total organic carbon/total organic nitrogen ratios (C/N); (e): *δ* ^13^ C_TOC_ (‰); (f): carbon mass accumulation rates (CMAR; g C m^−2^ yr^−1^) in 100‐year bins; (g): *δ* ^18^O_diatom_ record from Vydrino Shoulder piston‐core CON01‐605‐05 (Mackay *et al*., 2011); (h): four stacked records of relative abundance of haematite‐ stained grains (%HSG) in North Atlantic sediments (Bond *et al*., 2001); (i): dust concentrations (x10^3^ mL^−1^) from Qinghai–Tibetan Guliya ice core (Thompson *et al*., 1997); (j): 50‐year mean dust concentrations (mL^−1^) from Mount Kilimanjaro ice core NIF3 (Thompson *et al*., 2002) plotted on a log scale; (k): 50‐year mean dust concentrations (mL^−1^) from Huascarán ice core, Peru (Thompson *et al*., 2000) plotted on a log scale; (l): XRF Mn element density (cps) from Shaban Deep basin, northern Red Sea core GeoB 5836‐2 (Arz *et al*., 2006); (m): *δ* ^18^O (‰) of shallow‐water foraminifera *Globigerinoides ruber* from Shaban Deep basin, northern Red Sea core GeoB 5836‐2 (Arz *et al*., 2006); (n): dolomite (% wt) from Gulf of Oman sediment core M5‐422 (Cullen *et al*., 2000); (o): *δ* ^18^O (‰) of ostracod *Melanoides tuberculata* from palaeolake Kotla Dahar, NW India (Dixit *et al*., 2014); (p): *δ* ^18^O (‰) record from Mawmluh Cave speleothem, NE India (Berkelhammer et al., 2012); (q): *δ* ^18^O (‰) record from Dongge Cave speleothem, SE China (Dykoski *et al*., 2005). Light blue zones denote cold reversal events at c. 10.3, 8.2, 4.1 and 2.8 kyr bp.

Orbital configurations during the early Holocene resulted in very strong seasonality in central Asia (Bush, 2005); summers were warm and wet, while intensely cold winters contributed to low mean Northern Hemisphere temperatures (Marcott *et al*., 2013; Wanner *et al*., 2014; Fig. 3m). High early Holocene summer insolation (Fig. 3n) led to rapid melting of mountain glaciers and permafrost in southern Siberia (Groisman *et al*., 2013), and increased river flow into Lake Baikal (Mackay *et al*., 2011), resulting in lake levels rising by approximately 15 m (Urabe *et al*., 2004). High CMAR during the early Holocene (Fig. 3h) most likely represents allochthonous sources from melting permafrost, during summer months of high fluvial input (Fig. 6g); higher than average C/N (Fig. 6d) and *δ* ^13^C (Fig. 6e) values at this time are also indicative of increased allochthonous carbon to Lake Baikal sediments (Table 2).

**Table 2:** Factors likely to influence organic geochemistry in Lake Baikal sediments away from Holocene mean values: %TOC = 1.8%; CN = 11.6; *δ* ^13^C values = −29.03 ‰

Factor	TOC	C/N	*δ* ^13^C_ORG_
Increased planktonic diatoms	Increase	Decrease	Decrease^a^
Relative increase in pelagic productivity	Increase	Decrease	No change^b^
Relative increase in near‐shore productivity	Decrease	Unknown	Increase^c^
Increased picoplankton	Increase	Decrease	Unknown^d^
Increased terrestrial input from mature soils	Increase	Increase	Decrease^e^
Catchment DOM	No change	Increase	Increase^f^
Increased C_4_ terrestrial input^g^	NA	NA	NA
Increased atmospheric *p*CO_2_ h	No change	No change	No change
Increased ice cover^i^	Decrease	Unknown	No change
Gas hydrates^j^	No change	No change	No change

a At present, approximately 90% of organic matter in Lake Baikal is derived from phytoplankton, mainly diatoms during spring and autumn overturn; open water diatoms range between −28‰ and −35‰ (mean −29‰);

b In pelagic Baikal, the HCO_3_ pool is so large, no isotopic discrimination takes place (Yoshii *et al*., 1999);

c Flora in littoral regions have higher *δ* ^13^C values; aquatic macrophytes range between −5‰ and −18‰ and benthic algae between −5‰ and −11‰ (mean −9‰; Kiyashko *et al*., 1998; Yoshii, 1999; Yoshii *et al*., 1999);

d As far as we can ascertain, very little research has specifically looked at C fractionation in picoplankton. However, Sakata *et al*. (1997) suggest values of −22‰ to −30‰;

e Well‐developed soils result in an increase in ^13^C‐depleted respired CO_2_ (Hammarlund, 1992; Reuss *et al*., 2010);

f Dissolved organic matter from catchment rivers has *δ* ^13^C value of −26‰ to −27‰ (Yoshioka *et al*., 2002);

g Molecular isotopic stratigraphy of sedimentary long‐chain n‐alkanes did not detect any C_4_ plants within its watershed during the late Quaternary (Brincat *et al*., 2000);

h According to Prokopenko *et al*. (1999), increased Holocene atmospheric CO_2_ concentrations resulted in a decline in *δ* ^13^C_ORG_ values, but there is no relationship between Holocene CO_2_ concentrations and *δ* ^13^C_ORG_ values (Fig. 3);

i Biogenic silica inferred productivity is much lower during cold glacial periods with significantly extended ice cover (Mackay, 2007), but because of low overall primary production under the ice and higher CO_2_ solubility in colder water, isotopic discrimination is not thought to be important in Lake Baikal (Watanabe *et al*., 2004);

j A within‐lake process unique to Lake Baikal is the occurrence of sedimentary methane hydrates (Granin & Granina, 2002). Prokopenko & Williams (2004) suggested that the relatively negative Holocene TOC *δ* ^13^C values (in comparison with values for the late glacial of c. –24‰) may have been caused by deglacial methane emissions, with methane accumulating under winter ice (Prokopenko & Williams, 2005). However, teragrams of methane would need to be emitted, but only 10s of megagrams has actually been measured (Schmid *et al*., 2007), making it unlikely that *δ* ^13^C‐depleted methane drives lower sedimentary *δ* ^13^C values.

PC1 generally reflects vegetation responses to insolation‐driven changes in climate over the Holocene (Tarasov *et al*., 2007; Fig. 3m). Forest expansion mirrors the early Holocene decline in global CO_2_ concentrations (Fig. 3k) and an increase in ice core *δ* ^13^C (Fig. 3l) are indicative of the contribution made by expanding boreal forests to the global increase in terrestrial biomass (Elsig *et al*., 2009). Forest expansion will have led to stabilization of catchment soils which likely accounts for the significant negative relationship between PC1 and carbon burial rates after 9.6 kyr bp. Lower CMAR values may also be linked to lower Selenga River discharge at this time (Fig. 6g; Prokushkin *et al*., 2011).

#### Late Holocene

Scots Pine is a eurythermic and drought‐resistant conifer, and its maximum expansion between 7 and 4 kyr bp (Fig. 3b) is linked to regional summer temperature maxima and gradually increasing aridity in southern Siberia (Bush, 2005; Tarasov *et al*., 2007) caused by surface albedo feedbacks amplifying the climate system (Ganopolski *et al*. (1998). *δ* ^13^C_TOC_ values are lowest during this period, probably because pelagic diatoms dominate primary production at this time, as well as a potential contribution of respired carbon delivered to the lake from mature forest soils (Table 2). Increased CMAR at c. 5–4.5 kyr bp is coincident with a small peak in modelled summer relative humidity (Bush, 2005) and may be related to organic carbon from melting permafrost being delivered to the lake.

Declining late Holocene annual average air temperatures (Fig. 3m) are implicated in a renewed phase of Siberian permafrost formation on previously thawed surfaces, leading to characteristic two‐layered frozen structures (Anisimov *et al*., 2002). Renewed permafrost formation was likely responsible for persistent low carbon burial rates after 4 kyr bp (Fig. 6f). Persistent low CMAR observed here is in contrast to (i) mean CMAR for lakes in SW Greenland, which showed no difference between mid‐ and late Holocene periods (Anderson *et al*., 2009), and (ii) to mean CMAR for Chinese lakes which peaked between 3 and 1 kyr bp, linked to intensified human impact (Wang *et al*., 2015). These comparisons highlight the importance of regional activities when trying to understand delivery of allochthonous matter to lakes, although the potential influence of sediment focussing was not considered in either study.

### How do carbon dynamics respond to abrupt, sub‐Milankovitch scale events?

#### Early Holocene abrupt events

When ice sheets were still an important feature of North American and Eurasian landmasses, early Holocene climate was punctuated by pervasive millennial‐scale variability (e.g. Bond *et al*., 1997, 2001; Fisher *et al*., 2002; Mayewski *et al*., 2004; Wanner & Bütikofer, 2008; Wanner *et al*., 2008). Variability was associated with strong meltwater pulses flowing into the north Atlantic from melting Northern Hemisphere ice sheets (e.g. Bond *et al*., 1997; Carlson *et al*., 2008). These pulses resulted in atmospheric cooling (Rasmussen *et al*., 2006) which influenced terrestrial, freshwater and marine ecosystems worldwide through teleconnection processes (Björck *et al*., 1997; Mayewski *et al*., 2004; Berner *et al*., 2010; Smith *et al*., 2016). Modelling studies show that reductions in Atlantic meridional overturning circulation (AMOC) lead to northern surface wind anomalies in central Asia (Zhang & Delworth, 2005). The potassium (K^+^) record from the GISP2 ice core is a proxy for the strength of the Siberian High (SH). K^+^ records show that the SH was exceptionally intense at c. 10.8, 10.3, 9.2 and 8.2 kyr bp (Fig. 6b; Mayewski *et al*., 1997), periods coincident with reductions in AMOC. In East Asia, these events (together with changes in solar variability and ENSO) have been implicated in periods of weak Asian summer monsoon (e.g. D'Arrigo *et al*., 2005; Dykoski *et al*., 2005; Wang *et al*., 2005; Cai *et al*., 2008; Chen *et al*., 2015) and widespread aridity, for example, on the Tibetan Plateau (Thompson *et al*., 1997). Very little is known as to how these events impacted ecosystems in southern Siberia. During such events, a cooler Northern Hemisphere led to a strengthening of the Asian winter monsoon (Sun *et al*., 2012). We hypothesize that a more intense Siberian High resulted in a halt to the expansion of taiga forest and a reduction in active permafrost layers and caused a decline in pelagic productivity in the lake itself, linked to extended periods of ice and snow cover (Mackay *et al*., 2005).

Our data show that although significant changes in vegetation were occurring along the forest–steppe transition zone during the early Holocene (Fig. 4g), the direction of change (i.e. expansion of taiga forest) was unaltered, despite abrupt climate change events (Figs 3a, c and 6d). However, a small increase in steppe–forest index at 10.3 kyr bp (Fig. 3d) is concurrent with increases in steppe vegetation in the eastern Sayan Mountain range to the west of Lake Baikal (Mackay *et al*., 2012) and to the east of Baikal from Lake Kotokel (Bezrukova *et al*., 2010). We conclude therefore that insolation‐driven changes driving taiga forest expansion were stronger than sub‐Milankovitch forcings, although the latter did appear to result in temporary increases in steppe vegetation. The K^+^ peak at 10.3 kyr bp (Fig. 6b) was coincident with a significant decline in CMAR (Figs 3h and 4d) likely linked to both less permafrost melting and reduced river flow (less glacier melt) into the lake because of increased cold and aridity (Mackay *et al*., 2011; Fig. 6g). At this time, total diatom fluxes were highly variable (DCF; Figs 3i and 4e) with a significant increase in benthic diatom flux (Figs 3j and 4f), in line with impacts expected from changes in ice cover associated with a more intense Siberian High. These simultaneous, significant changes in both Lake Baikal and its catchment (Figs 4 and 6) highlight the importance of our analyses in unambiguously identifying the impacts of sub‐Milankovitch forcings on ecosystems remote from oceanic influences.

Although the 8.2 kyr event is one of most studied cold events linked to freshening of the North Atlantic, few, if any, high‐resolution records exist for its impact anywhere in Siberia (see fig. 1 in Morrill *et al*., 2013). In general, temperatures around the Europe and the North Atlantic cooled by approximately 1 °C, especially during wintertime (Alley & Ágústsdóttir, 2005; Rohling & Pälike, 2005), while there is strong evidence of increased aridity, especially in regions affected by the Asian monsoon (Morrill *et al*., 2013). A fall in Vydrino *δ* ^18^O_diatom_ values are indicative of reduced Selenga River flow (Fig. 6g), in line with increased aridity caused by a stronger Siberian High (Mackay *et al*., 2011), albeit a Siberian High not as strong as that which developed at 10.3 kyr bp (Fig. 6b). Even though we are able to reconstruct carbon dynamics at a resolution comparable to that required by Morrill *et al*. (2013) of under 50 years, any impact of increased cooling/aridity on regional ecosystems was minimal (Fig. 4). There is a small increase in the flux of benthic diatoms (Fig. 3j), but this is unlikely to be significant (Fig. 4f). Tentatively, therefore, our proxy data suggest that the 8.2 kyr event resulted in a small, temporary shift in the composition of primary producers in Lake Baikal, although overall carbon burial to the bottom sediments remained largely unchanged. Changes in vegetation composition in the southern Siberian catchment did not change either. That we observed no significant change in any of our analyses suggests that climatic impacts in southern Siberia were not as strong as experienced in regions around the, for example, North Atlantic. Perhaps, this is due to greater wintertime than summertime impacts (Alley & Ágústsdóttir, 2005), promoting aridity through a more prolonged Siberian High, but little change to summertime impacts such as diatom growth and permafrost melting.

#### Mid‐ to late Holocene abrupt events

Unlike early and late Holocene periods, it is not clear what caused mid‐Holocene cold events (Wanner *et al*., 2014). Nevertheless, the most striking change in all our geochemical indicators since the demise of Northern Hemisphere ice sheets occurs between 4.4 and 4.0 kyr bp (Figs 3 and 6). After this event, none of these indicators return to earlier Holocene values (Fig. 3), suggesting that a step change occurred with respect to carbon dynamics at the forest–steppe ecotone in southern Siberia.

The shift in carbon dynamics is coeval with abrupt hydrological changes reconstructed elsewhere in the world, linked to major shifts in large‐scale ocean‐atmosphere tropical dynamics, including a weakening of the El Niño Southern Oscillation (ENSO; McGregor *et al*., 2013; Dixit *et al*., 2014), and a weakening of the Asian summer monsoon (Dykoski *et al*., 2005; Wang *et al*., 2005; Berkelhammer *et al*., 2012). Increased aridity has also been reconstructed in Western Europe (Smith *et al*., 2016), the Middle East (e.g. Cullen *et al*., 2000; Arz *et al*., 2006; continental North America (Booth *et al*., 2005; Newby *et al*., 2014) and in northern Africa (Gasse, 2000). Kilimanjaro ice cover also declined at this time, and a 3 cm thick dust layer at c. 4 kyr bp is indicative of extremely dry conditions (Thompson *et al*., 2002; Fig. 6j). Dust records from ice cores on the Tibetan Plateau (Thompson *et al*., 1997) and tropical South America (Thompson *et al*., 2000) provide further evidence of widespread aridity at this time (Fig. 6i, k). It is likely therefore that the 4.1 kyr bp event in the Lake Baikal watershed may be due to a complex set of interactions between atmosphere and tropical ocean dynamics causing aridity in southern Siberia. In contrast, changes in diatom fluxes (Fig. 3i and j) were well within existing variability. Indeed, there were no significant changes observed in total diatom cell fluxes for the past 6 kyr in Lake Baikal (Fig. 4e), which suggests that factors that caused major fluxes in diatoms during the early Holocene had little influence during the second half of the interglacial.

Late Holocene cold events were caused by several ‘overlapping’ factors (such as volcanic eruptions and solar minima) against a backdrop of low NH summer insolation (e.g. Wanner *et al*., 2008, 2014) and amplified by centennial‐scale oceanic variability (Renssen *et al*., 2006). The event dated at c. 2.8 kyr bp is concurrent with a deep, abrupt reduction in solar activity (Fig. 6a; Grand Solar Minimum) which led to a decline in surface water temperatures in the North Atlantic (Andersson *et al*., 2003) and weaker meridional overturning circulation (Hall *et al*., 2004). A small increase in GISP2 K^+^ concentrations (Fig. 6b) indicates a strengthened Siberian High, concomitant with glacier advances in central Asia (Mayewski *et al*., 2004), a weaker Asian summer monsoon (Dykoski *et al*., 2005) and dust‐inferred aridity over the Tibetan plateau (Thompson *et al*., 1997; Fig. 6i). In the Lake Baikal region, the low resolution of *δ* ^18^O_diatom_ values at this time precludes robust interpretation of Selenga flow into Lake Baikal, except to say that it was likely low ((Fig. 6g). SiZer analyses reveal highly significant changes in carbon dynamics at this time (Fig. 4a–d), likely linked to a cooler, more arid climate. The increase in sedimentary *δ* ^13^C_TOC_ values (Fig. 3c) is concomitant with a small increase in benthic diatom fluxes, perhaps indicative of a relative shift in the balance between near and offshore primary producers at this time.

### How much carbon is stored in Lake Baikal sediments deposited during the Holocene?

Mean carbon burial rates for BAIK13‐7 for the past 50 years are 2.70 g C m^−2^ yr^−1^, similar to previous estimated rates in the south basin of 2.62 g C m^−2^ yr^−1^ (Müller *et al*., 2005) and 2.7 g C m^−2^ yr^−1^ (Alin & Johnson, 2007). Because of very high oxygen exposure times and the dominance of autochthonous sources (Sobek *et al*., 2009), these values are very much at the lower end of burial rates for lakes in general (Alin & Johnson, 2007) and northern, mid‐latitude (Heathcote *et al*., 2015) and culturally eutrophic (Anderson *et al*., 2014) lakes in particular. Values are similar, however, to long‐term mean rates for European (Kortelainen *et al*., 2004; Kastowski *et al*., 2011), high latitude (Anderson *et al*., 2009; Chinese (Wang *et al*., 2015) and other large oligotrophic lakes (Dean & Gorham, 1998; Einsele *et al*., 2001). The surface area of Lake Baikal covers 31 722 km^2^ (de Batist *et al*., 2006). Upscaling to the rate of organic carbon burial across the whole lake suggests that at least c. 8.56 × 10^−5^ Pg organic carbon are buried each year (similar to a previous estimate by Alin & Johnson (2007; 8.47 10^−5^ Pg C yr^−1^) but higher than that estimated by Einsele *et al*. (2001; 6.3 × 10^−5^ Pg C yr^−1^)). These rates suggest that 0.1–0.3% of estimated global annual storage of carbon into lake sediments (0.03–0.07 Pg C yr^−1^; Cole *et al*., 2007) occurs in Lake Baikal alone. In Europe, lakes are estimated to cover 240 000 km^2^ and sequester 1.25 Mt C yr^−1^ (Kastowski *et al*., 2011). Lake Baikal sequesters only about 7% of this amount, despite its area alone approximating to 15% of the surface area of all European lakes. That carbon burial rates in Lake Baikal are less than might be expected, is almost certainly down to its low burial efficiency.

Burial rates calculated for Lake Baikal were mainly obtained from the bottom sediments from the south basin. However, sedimentation is not continuous in these regions because large turbidite systems converge on the basin floors (Colman *et al*., 2003). The majority of palaeoenvironmental studies from Lake Baikal are undertaken in regions of continuous sedimentation such as interbasin or isolated highs, including the Academician Ridge and the Vydrino Shoulder (Fig. 1). It is from these two regions where the best resolved Holocene profiles, with available TOC data, can be found (e.g. Horiuchi *et al*., 2000; Watanabe *et al*., 2009; Fig. S4). A compilation of Holocene %TOC and *δ* ^13^C_TOC_ records reveals similarities across the length of the lake (Figs S3 and S4a,b). These temporally coherent observations indicate that regional‐scale drivers influenced carbon dynamics throughout Lake Baikal (Table 2; Fig. 5d). We therefore estimated organic carbon burial budgets during early (11.7–10 kyr bp, mid‐ (10–4 kyr bp) and late (4–1 kyr bp) Holocene periods. Burial rates of organic carbon were consistently higher at Vydrino than on the Academician Ridge, and mean burial rates were substantially higher during the early Holocene than the middle or late periods in both regions (Table 3). Burial rates are likely higher on the Vydrino Shoulder because, although autochthonous sources of organic carbon dominate both regions, burial efficiencies on the Academician Ridge are very low due to extraordinarily high oxygen exposure times of over 1000 years; on Vydrino oxygen exposure times are of the order of 10s of years (Sobek *et al*., 2009). There is considerable variation in burial rates between the two regions, but higher CMAR during the early Holocene highlights the importance of melting glaciers and permafrost on carbon budgets for the whole lake, not just coastal regions of the south basin. Using mean burial rates for early, mid‐ and late Holocene periods, we estimate that 1.03 Pg organic carbon have been buried in Lake Baikal sediments since the start of the Holocene, and almost one‐quarter of this was deposited before 10 kyr bp. Interestingly if we had just used annual rate of carbon burial for at BAIK13‐7 (2.7 g C m^−2^ yr^−1^), the estimated budget for buried carbon during the Holocene is similar at 1.00 Pg C. Global carbon storage in lake sediments during the Holocene range from 428 Pg (Cole *et al*., 2007) to 820 Pg (Einsele *et al*., 2001). Large lakes (area >10 000 km^2^) account for only 27 Pg C stored during the Holocene (Cole *et al*., 2007), so the Lake Baikal contribution to this figure is relatively minor (c. 4%). In comparison with Boreal lakes in general, Holocene carbon storage in Baikal sediments is still only between 4% and 5% (Kortelainen *et al*., 2004). Finally, we estimate that TOC buried in Lake Baikal sediments since its formation is likely to be substantially lower than the 4500 Pg given by Alin & Johnson (2007). They assumed constant sedimentation rates based on ^210^Pb dated cores from Edgington *et al*. (1991) of 0.0595 cm yr^−1^. However, these rates are from upper‐most sediments, and rates decline as sediments become more compacted. For the Holocene, we estimate average sedimentation rates of 0.0163 cm yr^−1^, while for other regions in the lake, sedimentation rates have been estimated to be about 0.030 cm yr^−1^ (Colman *et al*., 2003). Correcting for slower sedimentation rates in more compacted sediments, the total amount of organic carbon buried in Baikal sediments may well be in the order of only c. 2200 Pg carbon.

**Table 3:** Organic carbon burial rates determined for early, middle and late Holocene periods, based on five Holocene studies (see text for details and Fig. 1 for locations)

	Early Holocene CMAR (g C m^−2^ yr^−1^)	Middle Holocene CMAR (g C m^−2^ yr^−1^)	Late Holocene OC CMAR (g C m^−2^ yr^−1^)
CON01‐605‐5	8.97	6.21	3.84
Ver94.St16 (AR)	2.90	1.66	2.97
5GC (AR)	5.45	1.97	1.17
StPC (AR)	1.19	0.44	1.21
6GC (AR)	5.01	2.77	1.81
Mean (SD)	4.71 (2.94)	2.61 (2.18)	2.20 (2.17)

Although on a global perspective, Holocene carbon stored in Lake Baikal is relatively minor, that almost one‐quarter was deposited during the first few thousand years may have had major implications for biodiversity and ecosystem functioning of the lake. Large supplies of allochthonous carbon exported to lakes influence lake water properties including light and heat penetration because of the optical properties of dissolved organic matter (Solomon *et al*., 2015). For example, light extinction rates are faster, so resulting in a decline in primary production. These processes may account for the decline in diatom cell fluxes concomitant with rapid increases in CMAR (Fig. 3h and i). Work is ongoing to assess overall impact on diatom productivity–biodiversity relationships, and our unpublished results indicate a major decline in diatom palaeoproductivity at this time.

High‐resolution, multiproxy, palaeolimnology has demonstrated that carbon dynamics at the forest–steppe ecotone were highly variable during the Holocene. Allochthonous delivery was highest during the early Holocene because high summer insolation and increasing Northern Hemisphere temperatures caused rapid glacier retreat and melting permafrost, releasing carbon with little forest to stabilize catchment soils. We estimate that approximately one‐quarter of the Holocene carbon budget was sequestered during this period, which may have had a profound effect on primary production and diversity of large‐celled diatom species. Warm summers during the Early Holocene were vulnerable to extended winter cooling associated with periods of increased intensity of the Siberian High. These resulted in abrupt drops in organic carbon burial rates, concomitant with hydrological changes in the catchment. That these changes occurred almost simultaneously with changes elsewhere (e.g. decline in Asian summer monsoon (Dykoski *et al*., 2005) and increased aridity on the Tibetan Plateau (Thompson *et al*., 1997)) highlight that carbon dynamics in central Asia, far from oceanic influences, were highly responsive to changes in the global climate system during the early Holocene. Sustained low diatom productivity and carbon burial after c. 3 kyr bp is concurrent with the neoglacial, linked to pronounced cooling (Marcott *et al*., 2013) and aridity caused by vegetation and snow/ice albedo feedbacks in central Asia (e.g. Ganopolski *et al*., 1998; Renssen *et al*., 2006), leading to permafrost refreezing again.

Substantial warming over the past 50 years has led to permafrost degradation in southern Siberia (Törnqvist *et al*., 2014) and ecological changes in Lake Baikal (Hampton *et al*., 2014). Yet if current rates of permafrost warming are comparable to those during the early Holocene (Anisimov *et al*., 2002), the influence on carbon dynamics to Lake Baikal has yet to be realized. One reason for the discrepancy may be related to river discharge, which increases DOC input into Boreal lakes Prokushkin *et al*. (2011). During the early Holocene, river discharge into Lake Baikal was much greater (Mackay *et al*., 2011) because glaciers were melting, causing lake levels to rise substantially (Urabe *et al*., 2004), which in turn likely resulted in the very high carbon burial rates observed. In recent decades, average run‐off from Selenga River basin has declined, leading to decreased sediment loads (Törnqvist *et al*., 2014). Low mean Baikal carbon burial rates during the past 50 years are in contrast to other studies where recent increases in CMAR have been attributed to increased agriculture, for example China (Dong *et al*., 2012) and Europe (Anderson *et al*., 2014) or global warming/increased deposition of reactive nitrogen, for example northern lakes in North America (Heathcote *et al*., 2015). In the near future, it is doubtful whether nutrient enrichment or warming will result in increased carbon burial to Baikal sediments. There is increasing evidence that nutrient enrichment of coastal waters in Lake Baikal is starting to have an impact on near‐shore communities (Timoshkin *et al*., 2016), but there is as yet no evidence of nutrient enrichment in pelagic Lake Baikal (Izmest'eva *et al*., 2016). And although regional warming and forest fires are predicted to increase in the near future, driving the forest–steppe ecotone northwards (Tchebakova *et al*., 2009), southern Siberia is predicted to become more arid (Törnqvist *et al*., 2014), leading to a decline in Selenga River discharge. So despite further permafrost degradation, large quantities of released organic carbon may yet not find a route into Lake Baikal. Taken together, our data provide new and important insights into how abrupt climate change events can influence Holocene carbon dynamics in even very remote regions. However, understanding future changes to carbon dynamics must take account of hydrological variability as well as warming temperatures.

## Supporting information


**Figure S1.** PCA biplot of pollen data. Codes used include Cyp = Cyperaceae; AlnFrut = *Alnus fruticosa* type; Tubul = Compositae Asteroideae; PinSylv = *Pinus sylvestris* type; PinSib = *Pinus sibirica* type; Betnana = *Betula nana* type; Betun = *Betula* undifferentiated. Full details given in (Demske *et al*., 2005).

**Figure S2.** Breakpoint analysis of pollen PC1 data.

**Figure S3.** Compiled *δ* ^13^C data from Lake Baikal. A: Vydrino, this study; B: St. 5GC from the Academician Ridge (Watanabe *et al*., 2009); C: St.5PC from the Academician Ridge (Watanabe *et al*., 2009); D: St.6GC from the Academician Ridge (Watanabe *et al*., 2009); E: Ver94/St16 from the Academician Ridge (Horiuchi *et al*., 2000).

**Figure S4A.** Compiled TOC data from Lake Baikal plotted against a radiocarbon age scale. A: Vydrino, this study; B: St. 5GC from the Academician Ridge (Watanabe *et al*., 2009); C: St.5PC from the Academician Ridge (Watanabe *et al*., 2009); D: St.6GC from the Academician Ridge (Watanabe *et al*., 2009). E: Core Ver94.St.16 from the Academician Ridge (Horiuchi *et al*., 2000);

**Figure S4B.** Compiled Holocene TOC data from Lake Baikal plotted against a depth scale. A: Core Ver93/2‐GC24 from the Buguldieka Saddle, opposite the shallow waters of the Selenga Delta (Karabanov *et al*., 2004); B: Core BDP‐93‐2 from the Buguldieka Saddle, opposite the shallow waters of the Selenga Delta (Prokopenko *et al*., 1999). Approximate date horizons are derived from the revised chronology presented by Prokopenko *et al*. (2007), but no suitable age‐depth model is available from which to plot these up on an age scale.

**Table S1.** Data used to compile Fig. 1, including radiocarbon sample codes, depth intervals used to extract pollen for radiocarbon dating, details on pollen purity for radiocarbon analyses, uncalibrated and calibrated ages (calibrations based on IntCal13 radiocarbon calibration curve)
**Table S2.** Table shows key components of the datasets and transformations used in the GAM SiZer analyses. The term '*K*_max_' refers to the maximum value of the smoothing parameter k when calculating the GAM analyses.
