# Supplementary figures and images for: Holocene carbon dynamics at the forest–steppe ecotone of southern Siberia
Source: Glob Chang Biol. 2016 Dec 28;23(5):1942–60. doi: 10.1111/gcb.13583 (PMC6849524; doi:10.1111/gcb.13583)

## Supporting Information Fig 1

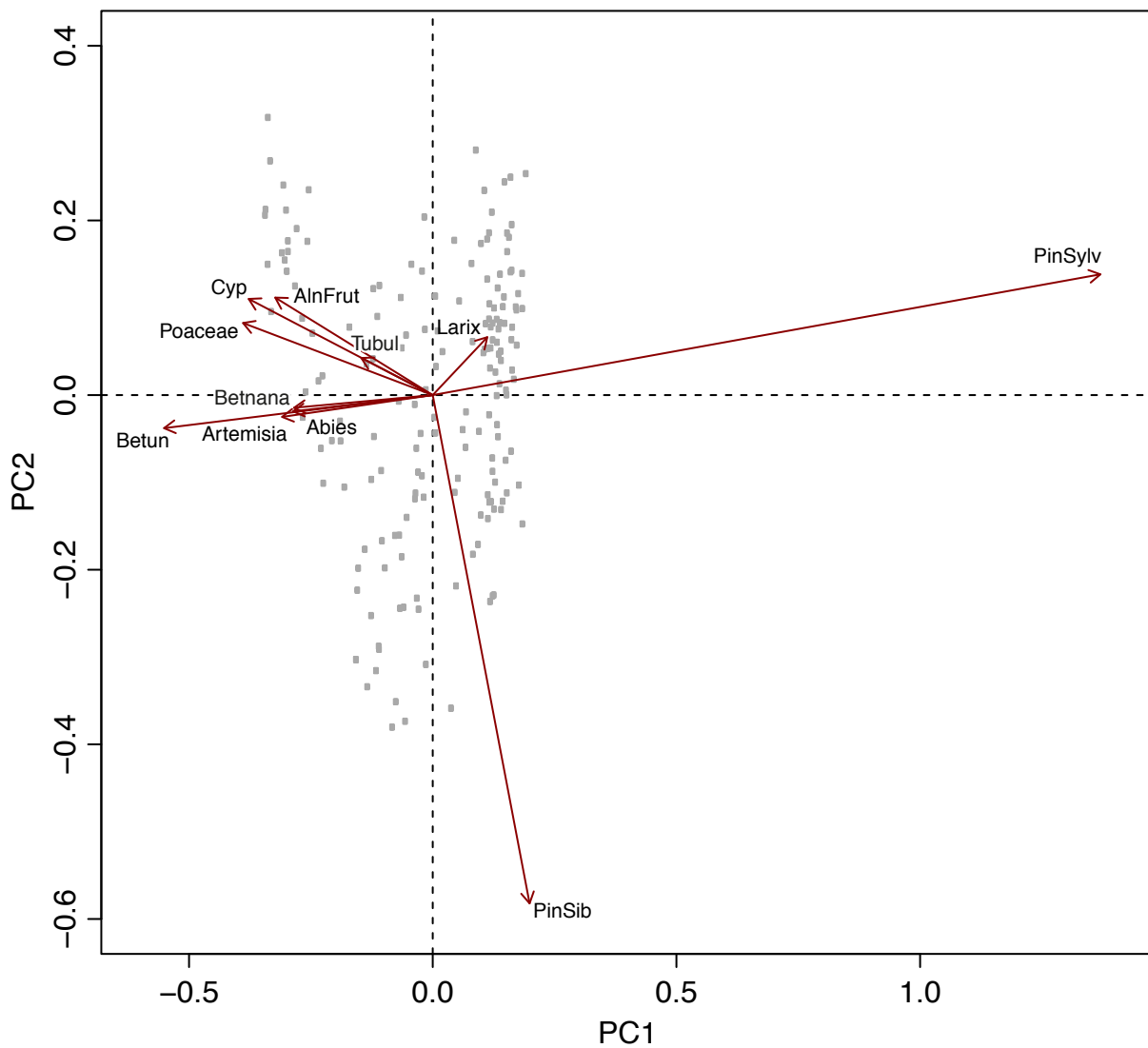

Supplement: Supplementary file 1 — Figure S1. PCA biplot of pollen data. Codes used include Cyp = Cyperaceae; AlnFrut = Alnus fruticosa type; Tubul = Compositae Asteroideae; PinSylv = Pinus sylvestris type; PinSib = Pinus sibirica type; Betnana = Betula nana type; Betun = Betula undifferentiated. Full details given in (Demske et al., 2005). [file GCB-23-1942-s001.pdf]

# Segmented LM

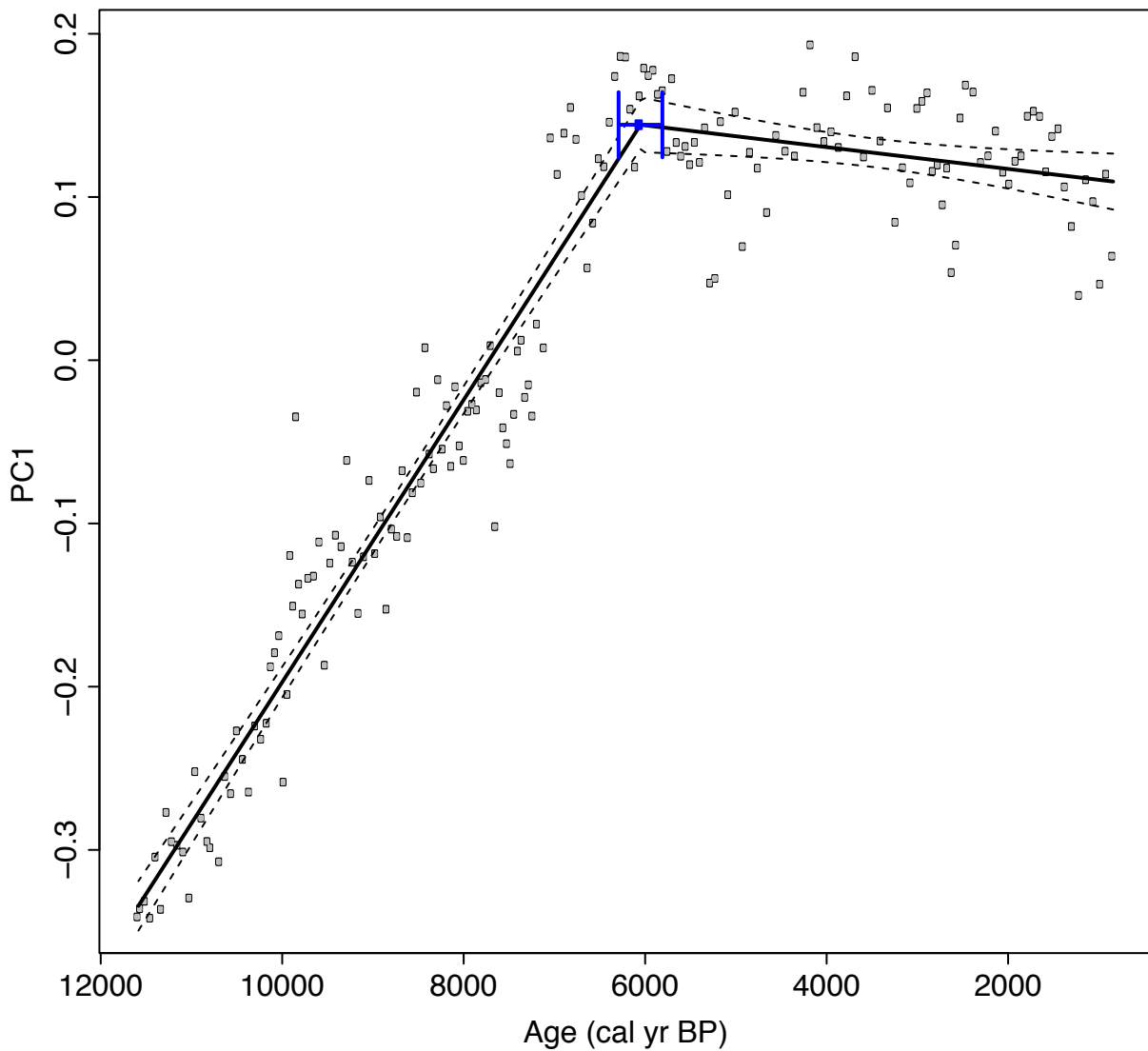

Supplement: Supplementary file 2 — Figure S2. Breakpoint analysis of pollen PC1 data. [file GCB-23-1942-s002.pdf]

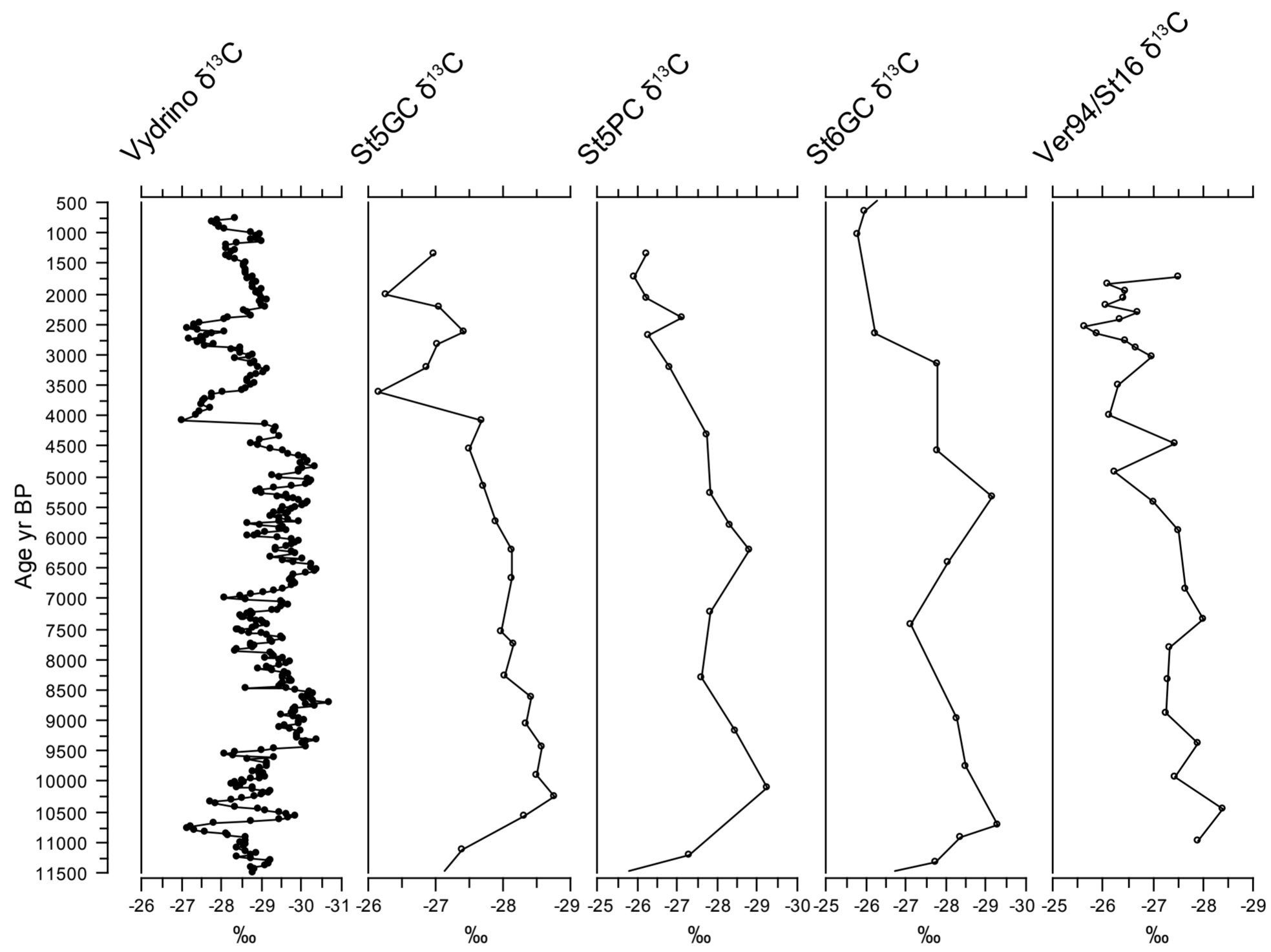

Supplement: Supplementary file 3 — Figure S3. Compiled δ 13C data from Lake Baikal. A: Vydrino, this study; B: St. 5GC from the Academician Ridge (Watanabe et al., 2009); C: St.5PC from the Academician Ridge (Watanabe et al., 2009); D: St.6GC from the Academician Ridge (Watanabe et al., 2009); E: Ver94/St16 from the Academician Ridge (Horiuchi et al., 2000). [file GCB-23-1942-s003.pdf]

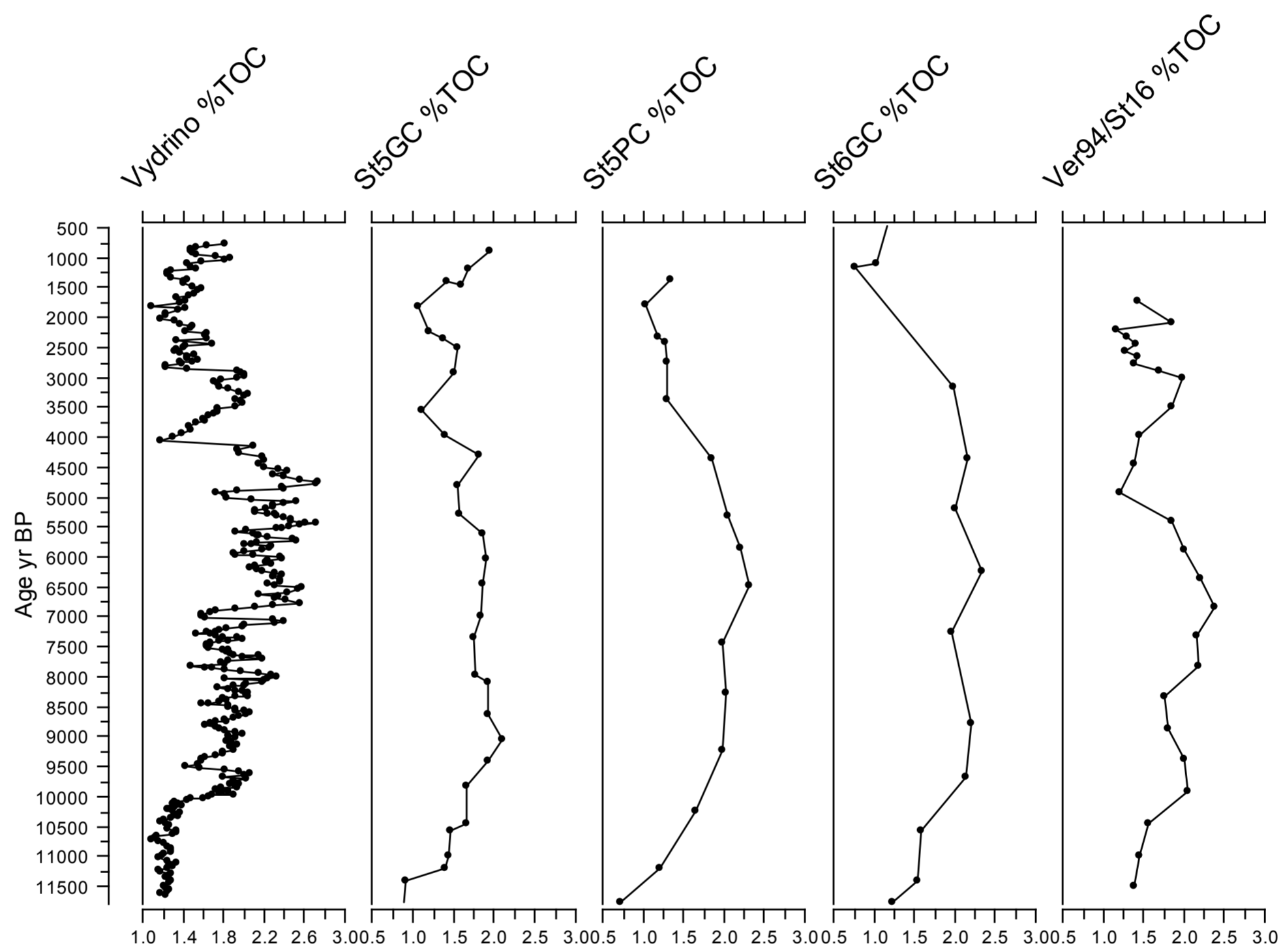

Supplement: Supplementary file 4 — Figure S4A. Compiled TOC data from Lake Baikal plotted against a radiocarbon age scale. A: Vydrino, this study; B: St. 5GC from the Academician Ridge (Watanabe et al., 2009); C: St.5PC from the Academician Ridge (Watanabe et al., 2009); D: St.6GC from the Academician Ridge (Watanabe et al., 2009). E: Core Ver94.St.16 from the Academician Ridge (Horiuchi et al., 2000); [file GCB-23-1942-s004.pdf]

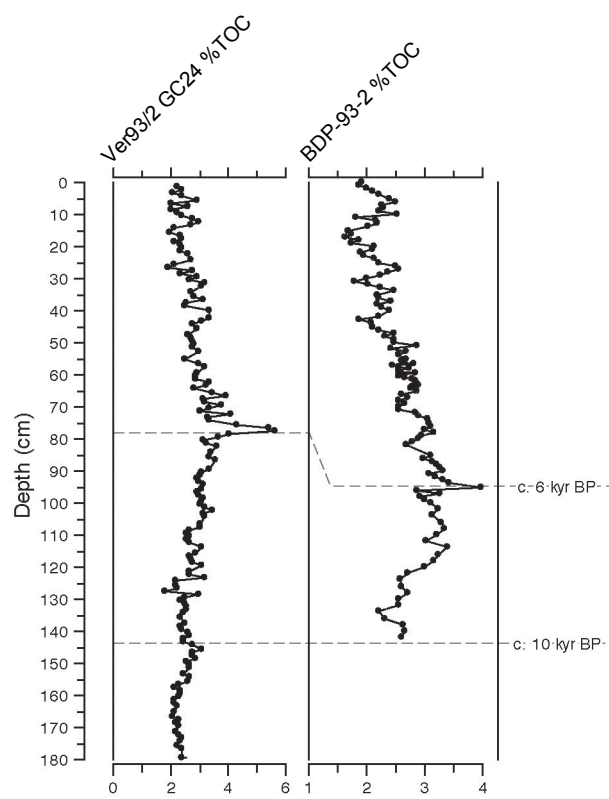

Supplement: Supplementary file 5 — Figure S4B. Compiled Holocene TOC data from Lake Baikal plotted against a depth scale. A: Core Ver93/2‐GC24 from the Buguldieka Saddle, opposite the shallow waters of the Selenga Delta (Karabanov et al., 2004); B: Core BDP‐93‐2 from the Buguldieka Saddle, opposite the shallow waters of the Selenga Delta (Prokopenko et al., 1999). Approximate date horizons are derived from the revised chronology presented by Prokopenko et al. (2007), but no suitable age‐depth model is available from which to plot these up on an age scale. [file GCB-23-1942-s005.pdf]
